# Assessment of different quantification metrics of [^18^F]-NaF PET/CT images of patients with abdominal aortic aneurysm

**DOI:** 10.1007/s12350-020-02220-2

**Published:** 2020-06-17

**Authors:** Mercy I. Akerele, Nouf A. Mushari, Rachael O. Forsythe, Maaz Syed, Nicolas A. Karakatsanis, David E. Newby, Marc R. Dweck, Charalampos Tsoumpas

**Affiliations:** 1 Biomedical Imaging Science Department, Leeds Institute of Cardiovascular and Metabolic Medicine, University of Leeds, Leeds, LS2 9NL UK; 2 British Heart Foundation Centre for Cardiovascular Science, University of Edinburgh, Edinburgh, UK; 3 Edinburgh Imaging Facility, Queen’s Medical Research Institute, University of Edinburgh, Edinburgh, UK; 4 Division of Radiopharmaceutical Sciences, Department of Radiology, Weil Cornell Medical College of Cornell University, New York, NY USA; 5 Biomedical Engineering & Imaging Institute, Icahn School of Medicine at Mount Sinai, New York, NY USA; 6 Invicro, London, UK

**Keywords:** Abdominal aortic aneurysm, spill-in effect, background correction, target-to-background ratio

## Abstract

**Background:**

We aim to assess the spill-in effect and the benefit in quantitative accuracy for [^18^F]-NaF PET/CT imaging of abdominal aortic aneurysms (AAA) using the background correction (BC) technique.

**Methods:**

Seventy-two datasets of patients diagnosed with AAA were reconstructed with ordered subset expectation maximization algorithm incorporating point spread function (PSF). Spill-in effect was investigated for the entire aneurysm (AAA), and part of the aneurysm excluding the region close to the bone (AAA_exc_). Quantifications of PSF and PSF+BC images using different thresholds (% of max. SUV in target regions-of-interest) to derive target-to-background (TBR) values (TBR_max_, TBR_90_, TBR_70_ and TBR_50_) were compared at 3 and 10 iterations.

**Results:**

TBR differences were observed between AAA and AAA_exc_ due to spill-in effect from the bone into the aneurysm. TBR_max_ showed the highest sensitivity to the spill-in effect while TBR_50_ showed the least. The spill-in effect was reduced at 10 iterations compared to 3 iterations, but at the expense of reduced contrast-to-noise ratio (CNR). TBR_50_ yielded the best trade-off between increased CNR and reduced spill-in effect. PSF+BC method reduced TBR sensitivity to spill-in effect, especially at 3 iterations, compared to PSF (*P*-value ≤ 0.05).

**Conclusion:**

TBR_50_ is robust metric for reduced spill-in and increased CNR.

**Electronic supplementary material:**

The online version of this article (10.1007/s12350-020-02220-2) contains supplementary material, which is available to authorized users.

## Introduction

Positron emission tomography/computed tomography (PET/CT) is a hybrid imaging technique that maximizes the information that can be extracted from both anatomical (CT) and functional (PET) images.^1^,^2^ While many radiotracers are used in PET/CT imaging, [^18^F]-fluorodeoxyglucose ([^18^F]-FDG) is the most common radiotracer; it can be used for different oncologic and non-oncologic applications. One of the non-oncologic applications of [^18^F]-FDG-PET/CT is for inflammatory vascular disease, as is the case with abdominal aortic aneurysms (AAAs).^3^ [^18^F]-FDG accumulation in the AAA region is related to an active inflammatory process, which can be defined as leukocyte infiltration in the adventitia, in addition to increased concentrations of circulating C-reactive protein.^4^,^5^ However, the role of [^18^F]-FDG in predicting the future growth and rupture risk of AAAs remains unclear, as studies have reported conflicting results.^6^,^7^ Moreover, local cellular hypoxia, which affects [^18^F]-FDG uptake and the contribution of the uptake from metabolically active adjacent structures, may confound the PET signal.^6^ Thus, more sufficient evidence is required to support the use of [^18^F]-FDG to predict future growth or rupture risk.

On the other hand, there is an increasing evidence of the efficacy of the sodium fluoride ([^18^F]-NaF) radiotracer as a marker of microcalcifications in AAAs, which can be a predictive sign of an increased risk of future rupture.^6^,^8^ Furthermore, [^18^F]-NaF PET/CT may be able to determine the hotspots of microcalcification. However, it is important to note that a major challenge and an inherent limitation of using [^18^F]-NaF for AAAs is that [^18^F]-NaF is taken up by the vertebrae because it is mainly a bone radiotracer.^9^,^10^ The anatomical site of the vertebrae is close to the posterior wall of the aorta, which results in an increased signal from this region because of the spill-in effect, leading to inaccurate quantification results.^11^ Thus, to increase the accuracy of the results, it is essential to either correct for the spill-in effect or else to identify the most appropriate quantification metrics which are less affected by the spill-in effect.

Spill-in correction can be applied during or after the standard OSEM reconstruction.^12^ It can be performed using different techniques, such as the background correction (BC) method in addition to PSF reconstruction.^13^-^15^ Although the PSF reconstruction method alone can correct for the generic partial volume effect, it has not been proven to be effective for the more specific spill-in correction when the region of interest is in close proximity to an active region.^15^-^17^ The PSF is modeled as a 3D Gaussian function, and it can be incorporated into the OSEM algorithm 13 where it is used in forward and backward projections.^13^,^15^ The BC method is applied after the background contribution to the PET-reconstructed image has been identified, using a segmented CT image as the background mask.^15^

After correcting for the spill-in effect, standardized uptake value (SUV) measurements can be derived where the spill-in effect, potentially, leads to a significant overestimation in SUV.^18^ This overestimation is partly influenced by the ROI selection criteria as a part of the active region might mistakenly be included with the target region, and past studies have shown that this spill-in effect is more prominent in SUV_max_ than the SUV_mean._^15^,^17^ Also, the spill-in effect reduces with iteration which comes at the expense of increased noise and reduced contrast.^19^ SUV_max_ is the highest voxel value within the region of interest (ROI); therefore, it is not so much affected by the ROI selection, but it is affected by noise and the spill-in effect.^20^-^22^ However, SUV_mean_ is the average of all the voxel values in the ROI; thus, while it is affected by the ROI selection, it is less sensitive to noise.^20^-^22^ SUV_max_ is the most common parameter used to measure radioactivity in patients, but SUV_mean_ is impractical and unreliable in atherosclerotic plaque quantification because it is affected by the ROI selection.^23^ It is also very difficult to define AAAs accurately because they do not have smooth edges 23; this leads to an inaccurate SUV_mean_. Furthermore, because of the limitations of both SUV_max_ and SUV_mean_, alternative SUV metrics can be derived in addition to SUV_max_ and SUV_mean_ which may be more robust to spill-in effect and noise. The present study proposed that the SUV metrics between SUV_max_ and SUV_mean_, such as SUV_90,_ SUV_70_ or SUV_50_, could possibly provide a better trade-off. These proposed metrics represent the mean of voxel values of the respective AAA region that are equal to or greater than 90%, 70%, or 50% respectively of SUV_max_.

However, when assessing vascular regions, the variability of the PET imaging protocols affects the SUV measurements.^23^ According to Huet et al.,^24^ the SUV values are influenced by several factors, such as image reconstruction, the number of iterations used and the post-filtering applied to the reconstructed images. The injected activity, the time between the injection and imaging of the patient and the acquisition duration have low variability, so they do not significantly affect the SUV values.^24^ This issue may limit the ability to conduct fair comparisons of the results from different institutions. To address this issue, the target-to-background ratio (TBR) was first introduced for assessing the atherosclerotic plaque.^25^ The TBR can be derived from SUV; TBR_max_, TBR_mean_, TBR_50_, TBR_70_ and TBR_90_ are derived from SUV_max_, SUV_mean_, SUV_50_, SUV_70_ and SUV_90_, respectively. The TBR is used to reduce the variation of the SUV measurements by correcting for the blood uptake.^23^ Therefore, TBR represents what the SUV actually represents, which is the measure of the radioactivity of the tracer in the vascular plaque. In the case of [^18^F]-NaF, the TBR represents plaque microcalcification. To date, no known studies have used TBR_50,_ TBR_70_ or TBR_90_ to measure radioactivity in atherosclerotic plaque, and no studies have made direct comparisons between different TBR metrics to determine the TBR metric that is most robust to the spill-in effect under specific circumstances.

Thus, the present study aims to compare a range of TBR metrics, including TBR_max_, TBR_90_, TBR_70_ and TBR_50_, to investigate which TBR might be more robust to the spill-in effect for use in [^18^F]-NaF AAA PET imaging. This comparison was performed using the standard reconstruction (including PSF modelling), and the correction (PSF+BC) methods at 3 and 10 iterations, and two different ROI delineations, to investigate which TBR metric is less sensitive to the spill-in effect, and for which method and iteration.

## Materials and Methods

### Study Datasets

For the present study, the data from 72 patients from the archive of the “Sodium Fluoride Imaging of Abdominal Aortic Aneurysms (SoFIA^3^)” study (NCT02229006)^11^ were used for this study. All participants were older than 50 years of age and were diagnosed with asymptomatic AAA. The aneurysms were measured using ultrasound undertaken at either the Royal Infirmary of Edinburgh, the Western Infirmary in Glasgow or the Forth Valley Royal Hospital, with an anteroposterior diameter of ≥ 4 cm for all patients whose data were used in the study. The data consist of 61 males and 11 females with age range 72.5 ± 6.9 years, body mass index 27.6 ± 3.5 kg/m^2^ and aortic diameter 48.8 ± 7.7 mm. The patients were injected intravenously using the 125 MBq of [^18^F]-NaF radiotracer. After 60 minutes of waiting uptake time, images were taken using a hybrid PET/CT scanner (Biograph mCT; Siemens Healthcare, Erlangen, Germany). Image acquisition was sequential starting with a low dose of the radiotracer and a 128-detector array CT scan, followed by PET imaging. During PET imaging, to ensure that the entire area of the aneurysm was covered, the acquisition was obtained from the thoracic aorta to the aortic bifurcation. This was achieved by applying three bed positions, each lasting 10 minutes.

Written informed consent was obtained from the participants to use their datasets, and approval was given by the research ethics committee in accordance with the Declaration of Helsinki.

### Image Reconstruction and Spill-in Correction

The datasets were reconstructed using the software for tomographic image reconstruction (STIR)^26^ with OSEM (21 subsets, 10 iterations). PSF reconstruction was incorporated into the reconstruction as an isotropic 3D Gaussian kernel with 4.4 mm full width at half maximum (FWHM) in both axial and transverse planes. The BC technique was used for spill-in correction.^15^ The bone was segmented from the CTAC image and the bone radioactivity was obtained from the reconstructed PET image (i.e., third iteration). This bone contribution was then included as an additive term in the reconstruction, producing a reconstructed image alleviating the contribution from the bone. Further details about the BC technique can be found in the literature.^15^,^17^,^27^ No post-filtering was applied to any of the reconstructed images.

### Datasets Analysis

Datasets were analyzed using “A Medical Imaging Data Examiner (AMIDE)” software^28^ in several steps. For region of interest (ROI) analysis, two ROIs were drawn on the CT images using the semi-automated ellipsoid method. One of the ROIs was defined as the entire aneurysm area, referred to in the present study as AAA. The other ROI included the entire aneurysm area, but excluded the posterior wall of the aorta that is near the vertebrae, referred to in this study as AAA_exc_. Following past research which showed that the spill-in effect is pronounced in regions within 2 voxels to the hot region,^15^ AAA_exc_ was drawn such that its distance from the bone is approximately 5mm, corresponding to about 2 voxels. The ROIs were then transferred to the reconstructed PET data. The standard clinical iteration is 3 iterations, but the image at 10 iterations was also used in the present study for comparison because a past work was found that the difference in uptake values due to the spill-in effect decreases by increasing the iterations and converges at approximately 10 iterations.^15^ Next, semi-quantitative measurements, which are the SUV metrics, were derived from the data including SUV_max_, SUV_90_, SUV_70_ and SUV_50_.

The TBR was calculated for each SUV metric by drawing a background ROI on the inferior vena cava for background blood pool correction. Consequently, the TBR for the two ROIs (AAA and AAA_exc_) for each SUV metric, method and iteration was calculated using Eq. (1):

1 $$ {\text{TBR}}_{i} = \frac{{{\text{SUV}}_{i} \left( {\text{Target}} \right)}}{{{\text{SUV}}_{\text{mean}} \left( {\text{Background}} \right)}} $$

where $i$ denotes max, 90, 70, and 50.

The effective spill-in effect from the bone into the aneurysm was quantified by the difference between the TBR AAA and TBR AAA_exc_ (DTBR) given by:

2 $$ {\text{DTBR}} = {\text{TBR}}_{\text{AAA}} - {\text{TBR}}_{{{\text{AAA}}_{\text{exc}} }} $$

The noise properties of the TBR metrics were evaluated using the contrast-to-noise ratio (CNR) given by:

3 $$ {\text{CNR}} = \frac{{{\text{TBR}}_{\text{AAA}} - {\text{TBR}}_{\text{Background}} }}{{\sqrt {{\text{SD}}_{\text{AAA}}^{2} + {\text{SD}}_{\text{Background}}^{2} } }}. $$

### Statistical Analysis

Statistical analyses were undertaken using IBM SPSS statistics software package, version 23. For all patients, the difference in the TBR metrics (max, 90, 70 and 50) were compared between the two reconstruction methods and iterations using paired t-test. The statistical analyses were performed with a 95% confidence interval (CI), and a *P*-value of ≤ 0.05 was considered to be statistically significant.

## Results

**Fig. 1:**
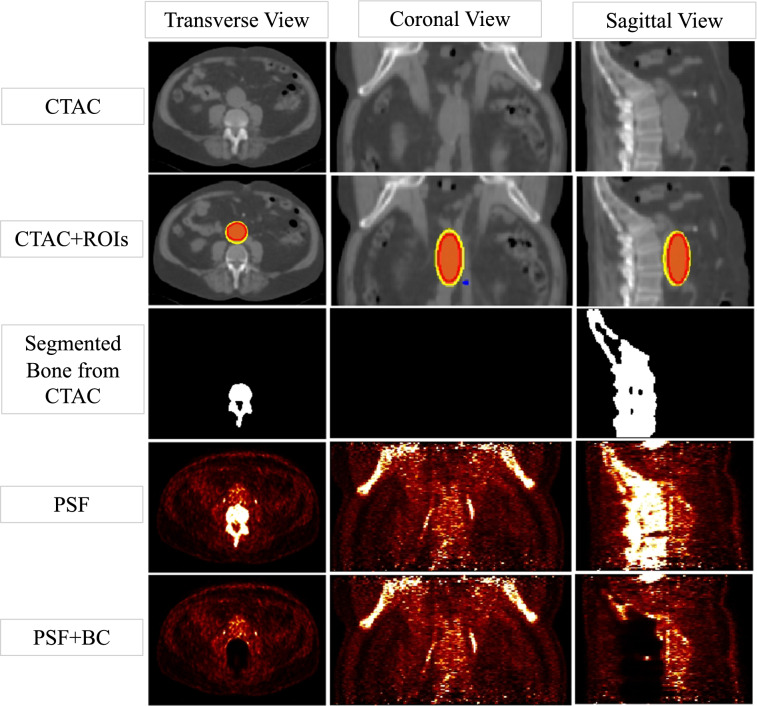
CT images and PET-reconstructed images of a patient dataset, showing a high [^18^F]-NaF uptake in the bone and the aneurysm. The activity contribution from the bone was removed using PSF+BC. The ROIs used to extract the SUVs at the aneurysm are shown on the CTAC image. The outer yellow and inner red ROIs represent AAA and AAA_exc_, respectively. Following past research,^15^ AAA_exc_ was drawn such that its distance from the bone is approximately 4 mm. The blue small spherical region highlights the background ROI used for blood pool correction and the calculation of TBR

Figure 1 shows a sample CTAC and the PET-reconstructed images from a patient which indicate a high [^18^F]-NaF uptake in the aneurysm and the bone. The segmented bone used for the BC is also shown. Note that the bone uptake contribution including the spill-in has been removed in the PSF+BC image.

**Fig. 2:**
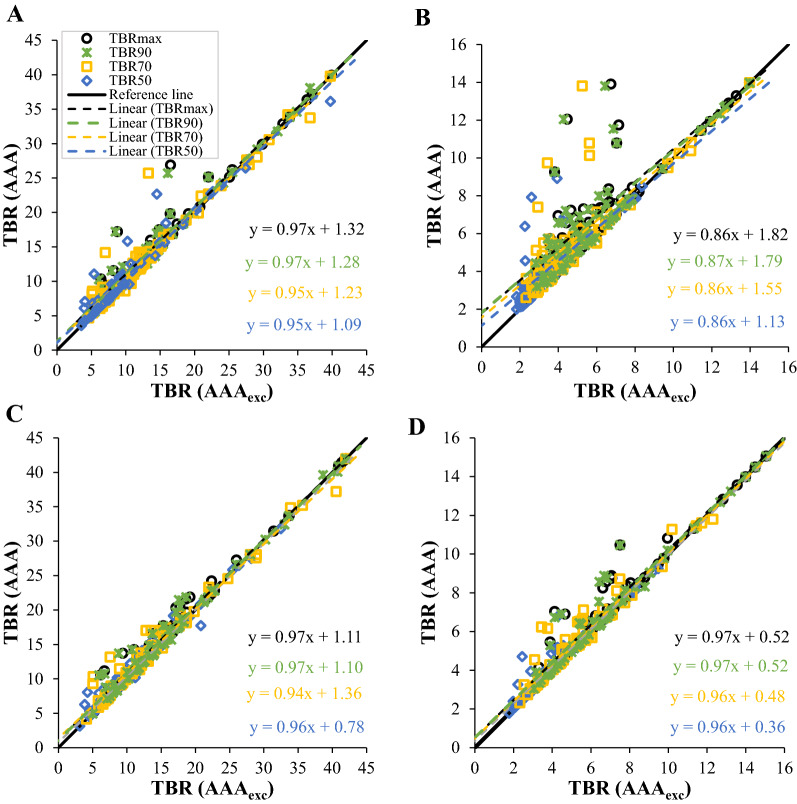
Comparisons of the different TBR metrics using the two ROI delineations. (**A**) and (**B**) show the PSF at 10 and 3 iterations, respectively, while (**C**) and (**D**) show the PSF+BC at 10 and 3 iterations, respectively

Figure 2 shows the comparisons of the TBR metrics for different ROIs, methods, and iterations. As seen for PSF at 3 iterations (Figure 2b), the different TBR metrics form variations above the black reference line when comparing the values of TBRs for the two ROIs (AAA and AAA_exc_). Because the reference line indicates that the difference between the TBR values of the *X*-axis (i.e., AAA_exc_) and *Y*-axis (i.e., AAA) equals zero, variations above the reference line indicate that, for PSF at 3 iterations, TBR AAA is higher than TBR AAA_exc_. Moreover, TBR_50_ was the closest metrics with the lowest intercept to the reference line, followed by TBR_70_, then TBR_90_ and TBR_max_. Higher uptake values can be seen in the AAA ROI, where the highest values were recorded for TBR_max_, TBR_90_ and TBR_70_. While TBR_50_ also had some high values, they were however closer to the reference line. The plot of PSF at 10 iterations (Figure 2a) shows that the variations between the TBR metrics decreased and then became closer to the reference line, with almost similar intercepts. While there are some high uptake values for TBR_max_, TBR_90_, TBR_70_ and TBR_50_ at the AAA ROI, TBR_50_ had lower values than the other TBRs.

For PSF+BC at 10 iterations (Figure 2c), the TBR metrics became closer to each other; therefore, their divergence was reduced. TBR_70_ intercept was the farthest from the reference line. The plot of PSF+BC at 3 iterations (Figure 2d) shows that the TBR metric lines were similar to the results obtained for PSF+BC at 10 iterations; they had intercepts closer to the reference line, and the TBR line extensions were almost identical to the reference line. The dispersion of the TBRs values was slightly higher for the PSF+BC at 3 iterations than at 10 iterations, especially for TBR_70_, TBR_90_ and TBR_max_. However, this dispersion is minimal when compared with PSF at 3 iterations. Furthermore, all values of different TBR metrics for PSF+BC were close to the reference line in comparison to the values for PSF. TBR_50_ was the closest to the reference line, while TBR_70_, TBR_90_ and TBR_max_ had a slightly higher uptake at the AAA ROI.

It could also be seen that the scattering of the TBR values was greater in the PSF method than the PSF+BC method, especially at 10 iterations, and the highest intercept values were for TBR_max_, TBR_90_ and TBR_70_, while the TBR_50_ values were closer to the reference line. However, as seen in Table 1, for the PSF method, the differences between the iterations were not statistically significant for all the TBR values except for TBR_70_ with *P*-value 0.04. In addition, the PSF+BC method had similar statistical results, except for TBR_max_ and TBR_90_ with *P*-values equal to 0.002 and 0.04, respectively.

**Table 1:** Paired t-test analysis results showing the *P*-values of the difference in TBR metrics between methods and for each iteration

	10 iterations	3 iterations	PSF	PSF+BC
	PSF vs PSF+BC	PSF vs PSF+BC	10 vs 3 iteration	10 vs 3 iteration
TBR_max_	0.33	0.0002*	0.31	0.002*
TBR_50_	0.32	0.0006*	0.82	0.11
TBR_70_	0.99	0.002*	0.04*	0.06
TBR_90_	0.25	0.0003*	0.25	0.04*

To further evaluate the TBR metrics and their robustness to spill-in effect and noise reduction, the differences in TBR between AAA and AAA_exc_ (i.e., DTBR) was plotted against the CNR as shown in Figure 4. As expected, the difference in TBR due to the different ROI delineation was high at lower iteration but reduces as iteration increases. However, this comes as the expense of reduced CNR. TBR_90_ has the highest CNR but the DTBR was high just like TBR_max_. TBR_50_ gave the best trade-off between increased CNR and reduced DTBR

**Fig. 3:**
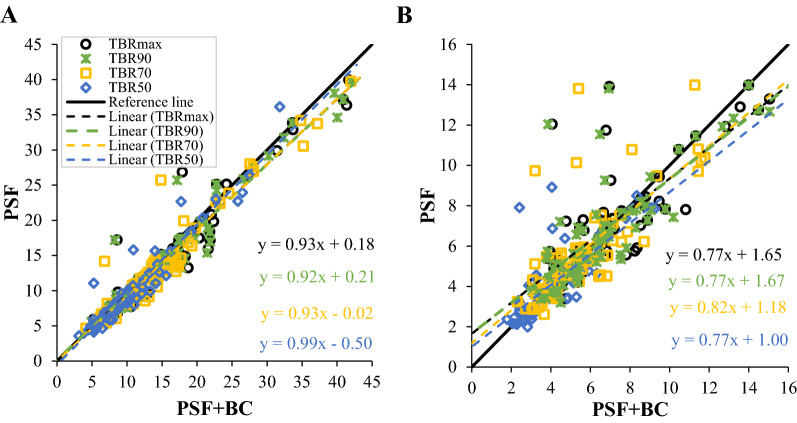
Comparison of the TBR metric for PSF and PSF+BC at (**A**) 10 iterations, and (**B**) 3 iterations

Additional analyses were conducted to compare the methods and iterations to investigate the differences in the TBR metrics. Figure 3 shows the comparisons of the different methods (PSF vs PSF+BC) at 3 and 10 iterations. At 10 iterations, there was a variation between the TBR metric lines. The TBR lines closest to the reference line were TBR_50_, followed by TBR_70,_ TBR_max_ and TBR_90_. The closest high values to the reference line were for TBR_50_. However, as shown in Table 1, no statistically significant difference was found between PSF and PSF+BC for all the TBR metrics at 10 iterations.

On the other hand, at 3 iterations, as seen in Figure 3, the variations between the TBR metrics lines was greater than the variations in the lines at 10 iterations, and the lines were far from the reference line. The TBR values were higher for the PSF method, with an increase in the number of values away from the reference line. As previously shown, the highest values were for TBR_70_, TBR_90_ and TBR_max_, while TBR_50_ had few anomalous values in relation to the reference line. As seen in Table 1, the comparison of PSF vs PSF+BC for all the TBR metrics indicates a statistically significant difference between the methods at 3 iterations, with *P*-values equal to 0.0002, 0.0006, 0.002, and 0.0003 for TBR_max_, TBR_50_, TBR_70_ and TBR_90_, respectively.

## Discussion

Despite the increasing evidence of the efficacy of the sodium fluoride ([^18^F]-NaF) radiotracer as a marker of microcalcifications in AAAs,^6^,^8^ a major confounding issue is the spill-in contamination from the bone (where the tracer is taken up) to the adjacent aneurysm.^9^,^10^ Our previous study^17^ extensively investigated the spill-in effect in [^18^F]-NaF PET imaging of AAA, and it was shown that the spill-in effect depends on the activity uptake in the bone, proximity of the aneurysm to the bone, as well as ROI delineation criteria. This effect poses a great challenge to the quantification accuracy at the aneurysm site and it may adversely affect AAA disease prediction and patient management.^11^,^17^ As reflected by the SOFIA^3^ study,^11^ better AAA disease prediction using [^18^F]-NaF, in addition to clinical risk factors including AAA diameters, would be of great benefit to patients with high-risk aneurysms which size may be smaller than what the current guidelines may suggest (i.e., 55 mm). Thus, to increase the accuracy of the AAA quantification, it is essential to either correct for the spill-in effect or else to identify the most appropriate quantification metrics which are less affected by this effect. This was the main aim of the study.

The present study investigated TBR metrics using PSF and PSF+BC methods, 3 and 10 iterations and two semi-automated ROIs (AAA vs AAA_exc_) to determine which TBR metric is less sensitive to the spill-in effect coming from the hot region (i.e., bone) adjacent to the aneurysm. TBR_mean_ is impractical for quantifying uptake at the aneurysm due to the heterogenous activity distribution in the aneurysm, and ill-definition of the aneurysm edges. Therefore, the use of the semi-automated method for ROI definition may result in inaccurate TBR_mean_, thus it was excluded from this study.

By comparing the TBR values in different situations, and observing the results shown in Figure 2, it can be concluded that the more the iterations, the more robust the TBR values, and these values do not appear to be affected by the ROI. Increasing the iterations for the same method reduces the difference in the uptake values of the two ROIs. Because the uptake values consistently increase (for PSF+BC) or decrease (for PSF) while increasing the number of iterations until they reach convergence,^15^,^29^ which may explain the consistency of the TBRs values at 10 iterations. This result is the focus of attention because the difference in how individuals draw the ROI may become less important by increasing the number of iterations. Furthermore, by applying 10 iterations, the TBR results indicated that both methods were similar. However, Akerele et al.^15^ noted that, although increasing the number of iterations reduces the impact of the spill-in effect, it also increases noise and decreases the contrast-to-noise ratio.

By comparing the two methods (PSF and PSF+BC), as seen in Figure 3 and Table 1, the TBRs are more consistent and less sensitive to the spill-in effect with the PSF+BC method. Thus, the TBRs in both iterations appear to have converged. This may indicate the importance of applying PSF+BC, because it contributes to minimizing the impact of the spill-in effect. It might be better to use PSF+BC at 3 iterations, because its behavior is very similar at 3 and 10 iterations, rather than increasing the number of iterations for the PSF method, due to the increase in noise. The results for the PSF method conflict with the findings reported in the literature review where PSF, theoretically, can provide an advantage in terms of reducing TBR overestimation due to the spill-in effect.^30^-^33^ PSF alone is used as a correction for the generic partial volume effect, but it has not been proven to be effective for the more specific spill-in correction, as is the case with AAA assessment. Moreover, Akerele et al.^15^ reported that for proximal lesions to an active region, incorporating PSF into the standard OSEM reconstruction has no added advantage compared to using OSEM alone. However, this could be due to the fact that only a simple space invariant PSF was used.

**Fig. 4:**
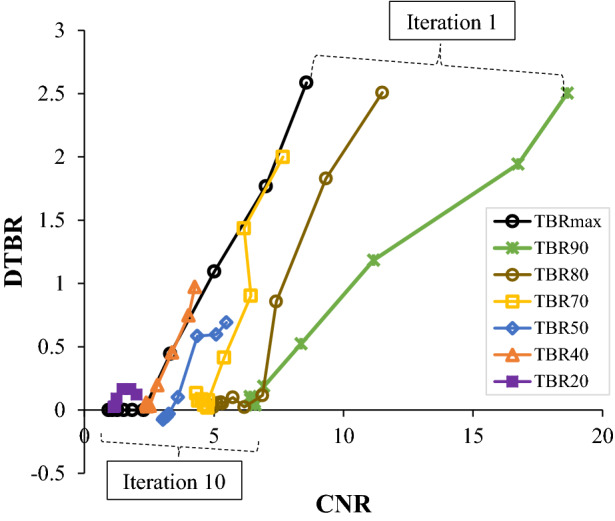
The plot of difference in TBR values for AAA and AAA_exc_ (DTBR) against CNR for all the TBR metrics as iteration increases. This is shown for a sample patient reconstructed with PSF. A robust TBR metric will show low DTBR and high CNR

In terms of the robustness of the TBR metrics to noise and spill-in reduction, the graph of DTBR against CNR (Figure 4) shows that for each TBR metrics, the difference in TBR due to the different ROI delineation was high at lower iteration but reduces as iteration increases. However, this comes as the expense of reduced CNR. TBR_90_ has the highest CNR but the DTBR was high just like TBR_max_. TBR_50_ gave the best trade-off between increased CNR and reduced DTBR. Overall, TBR_50_ appears to be the most robust TBR value as it is less affected by the ROI and the spill-in effect for both PSF methods (with and without correction) and all iterations, followed by TBR_70_; in contrast, the closer the TBR was to the TBR_max_, the more it was affected by the spill-in effect.

The present study’s TBR findings are consistent with the results reported by Boucek et al.^34^ who investigated the accuracy of SUV_max_ in a tumor response assessment and found that the SUV_max_ was influenced by the spill-in effect. Furthermore, Visser et al.^35^ stated that the impact of the spill-in effect can be reduced using voxel values equal to or greater than a fixed percentage of the SUV_max_, which is similar to what was used in the present study: TBR of SUV_50,_ SUV_70_ or SUV_90_. Because the TBR values were derived from the SUV values, these two studies can be considered to have similar results, which, in turn, might support the results of the present study. However, further studies are needed to investigate the TBRs results to obtain fair comparisons, because the SUV metrics differ from one center to another depending on several factors that are difficult to standardized due to the differences between scanners, image reconstruction and data analysis software.^24^ It is worth noting that the recommendations in this work are rather task-based. If one is concerned mainly about quantification accuracy, then it is recommended to use more iterations with or without BC. This is because the more the iterations, the better the quantification and the less the spill-in effect. However, if better contrast and lesion detectability is of utmost importance, it is best to use less iteration and then apply the BC method.

## Study Limitations and Future Work

The results of the present study are subject to some limitations. First, drawing ROIs using the semi-automated method could have affected the measurement of the TBRs; the manual method might be more accurate for determining the size of the aneurysm because the AAA wall is not often well defined. Therefore, an issue that was not addressed in this study was whether or not the semi-automated method differs from the manual method in terms of TBR measurement accuracy. Second, only two iterations (i.e., 3 and 10) were used to extensively investigate the impact of increasing iterations on the spill-in effect rather than evaluating many more iterations. Finally, no known studies have made direct comparisons between different TBR metrics, which prevented the ability to compare the study’s TBRs results to other studies. Therefore, it is recommended that future studies be conducted to further explore the current topic.

Also, this study needs to be further validated with larger cohort to potentially distinguish any differences between male and female AAA patients. The main reason that we had more male AAA patients than females in this study is the fact that the male sex is one of the risk factors for AAA.^36^,^37^ So, our study represents a typical AAA cohort with larger number of male (*N* = 61) than female (*N* = 11) patients. However, there might be some sex-specific variables such as arteries sizes which might affect the generalization of our results. Furthermore, it is interesting to investigate whether there is a significant difference in aneurysm shape and heterogeneity of [^18^F]-NaF uptake between male and female patients, which may impact ROI thresholding as proposed in this study.

Although the application of the BC technique helped to reduce the spill-in effect, there are several other challenges and biases which could affect the TBR results such as the scanners, image reconstruction algorithms and data analysis software used across clinical centers.^38^ So, there is a need to further investigate other metrics that could have more accurate results than SUV metrics or TBR metrics. Advanced metrics, known as radiomics, have emerged and may help overcome the limitations of using SUV and TBR metrics. Radiomics can provide more reliable prognostic information than conventional SUV metrics.^39^ Several studies have compared radiomics and SUV in terms of therapy outcome, and the results favored the use of radiomics.^40^-^44^ Therefore, extensive research should be conducted to assess the reliability and robustness of these advanced metrics before they are clinically adopted.

## New Knowledge Gained

In this study, we have shown that the most commonly employed quantification metric of TBR_max_ for clinical assessment in [^18^F]-NaF PET/CT imaging of AAA is prone to quantification overestimation, partly due to the spill-in effect from the bone into the aneurysm, and also due to differences in ROI delineation criteria. The use of lower TBR thresholds can yield more robust [^18^F]-NaF quantification that is less sensitive to spill-in effects, with TBR_50_ resulting in the least overestimation.

## Conclusions

The quantitative metric of TBR contrast in AAA regions of [^18^F]-NaF images acquired from human PET/CT exams appeared to be less sensitive to the spill-in effect when using PSF+BC and/or when increasing the number of OSEM iterations. However, the noise levels increased with the number of OSEM iterations thus reducing CNR and potentially impacting AAA lesions detectability. Therefore, to enhance [^18^F]-NaF quantification in AAA, we recommend applying the PSF+BC method with few iterations. Moreover, the use of a 50% relative-to-maximum threshold for defining the TBR (TBR_50_) was found to be most robust metric as it exhibited the lowest sensitivity to the spill-in effect; in contrast, the closer the TBR definition was to the TBR_max_, the more it was affected by the spill-in effect.

## Electronic supplementary material

Below is the link to the electronic supplementary material.

Supplementary material 1 (PPTX 1858 kb)

## Abbreviations

PET/CT: Positron emission tomography/computed tomography
OSEM: Ordered subset expectation maximization
3D: Three-dimensional
PSF: Point spread function
[^18^F]-FDG: [^18^F]-fluorodeoxyglucose
AAA: Abdominal aortic aneurysm
[^18^F]-NaF: Sodium fluoride
BC: Background correction
SUV: Standardized uptake value
ROI: Region of interest
TBR: Target-to-background ratio
STIR: Software for tomographic image reconstruction
AMIDE: A medical imaging data examiner
