# Supplementary material for: Assessment of different quantification metrics of [18F]-NaF PET/CT images of patients with abdominal aortic aneurysm
Source: J Nucl Cardiol. 2020 Jun 17;29(1):251–61. doi: 10.1007/s12350-020-02220-2 (PMC8873073; doi:10.1007/s12350-020-02220-2)
Supplement: Supplementary file 1 — Supplementary material 1 (PPTX 1858 kb) [file 12350_2020_2220_MOESM1_ESM.pptx]

## Slide 1
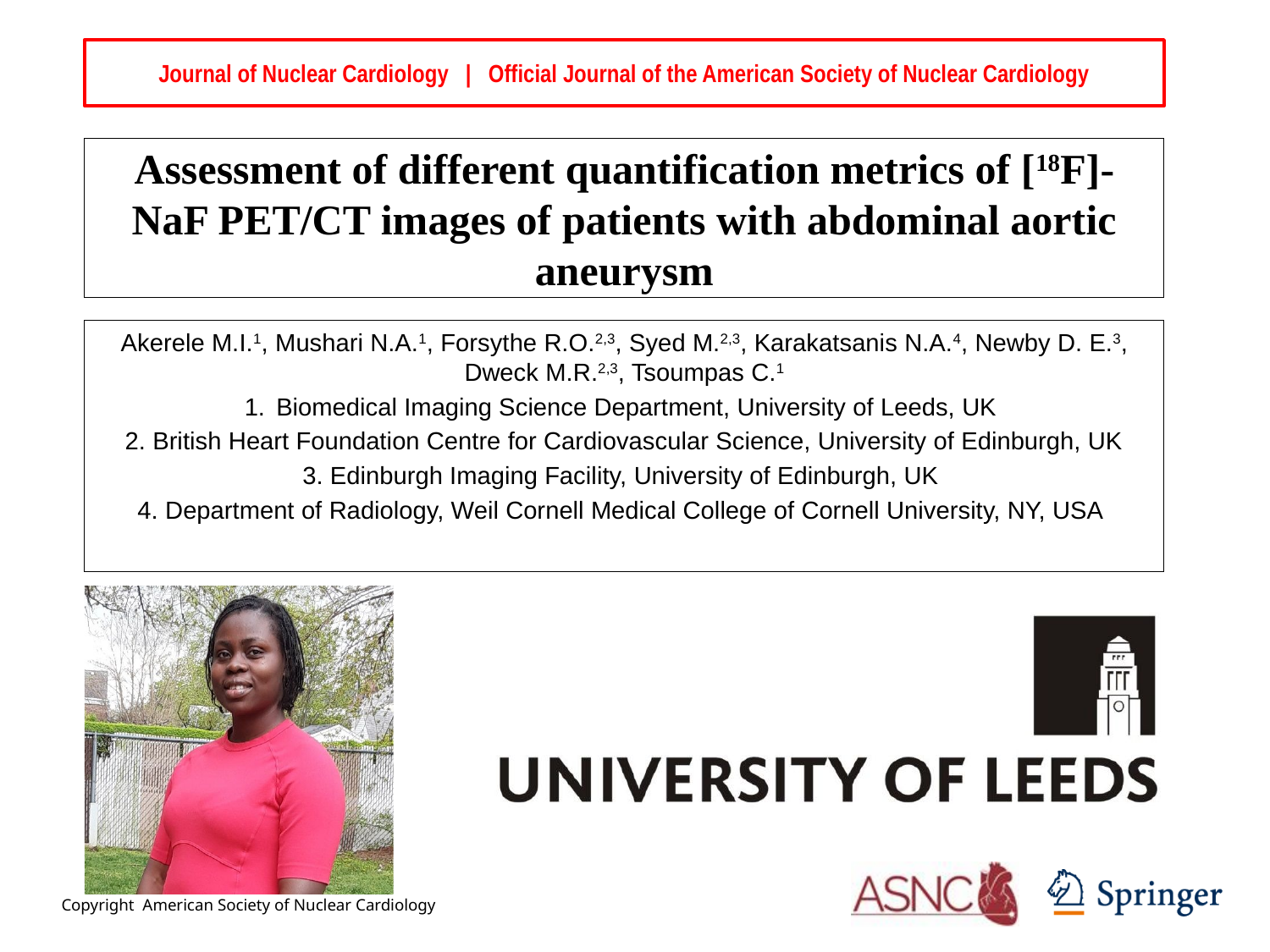

Journal of Nuclear Cardiology | Official Journal of the American Society of Nuclear Cardiology
# Assessment of different quantification metrics of [18F]-NaF PET/CT images of patients with abdominal aortic aneurysm
Akerele M.I.1, Mushari N.A.1, Forsythe R.O.2,3, Syed M.2,3, Karakatsanis N.A.4, Newby D. E.3, Dweck M.R.2,3, Tsoumpas C.1
Biomedical Imaging Science Department, University of Leeds, UK
2. British Heart Foundation Centre for Cardiovascular Science, University of Edinburgh, UK
3. Edinburgh Imaging Facility, University of Edinburgh, UK
4. Department of Radiology, Weil Cornell Medical College of Cornell University, NY, USA
Copyright American Society of Nuclear Cardiology

## Slide 2
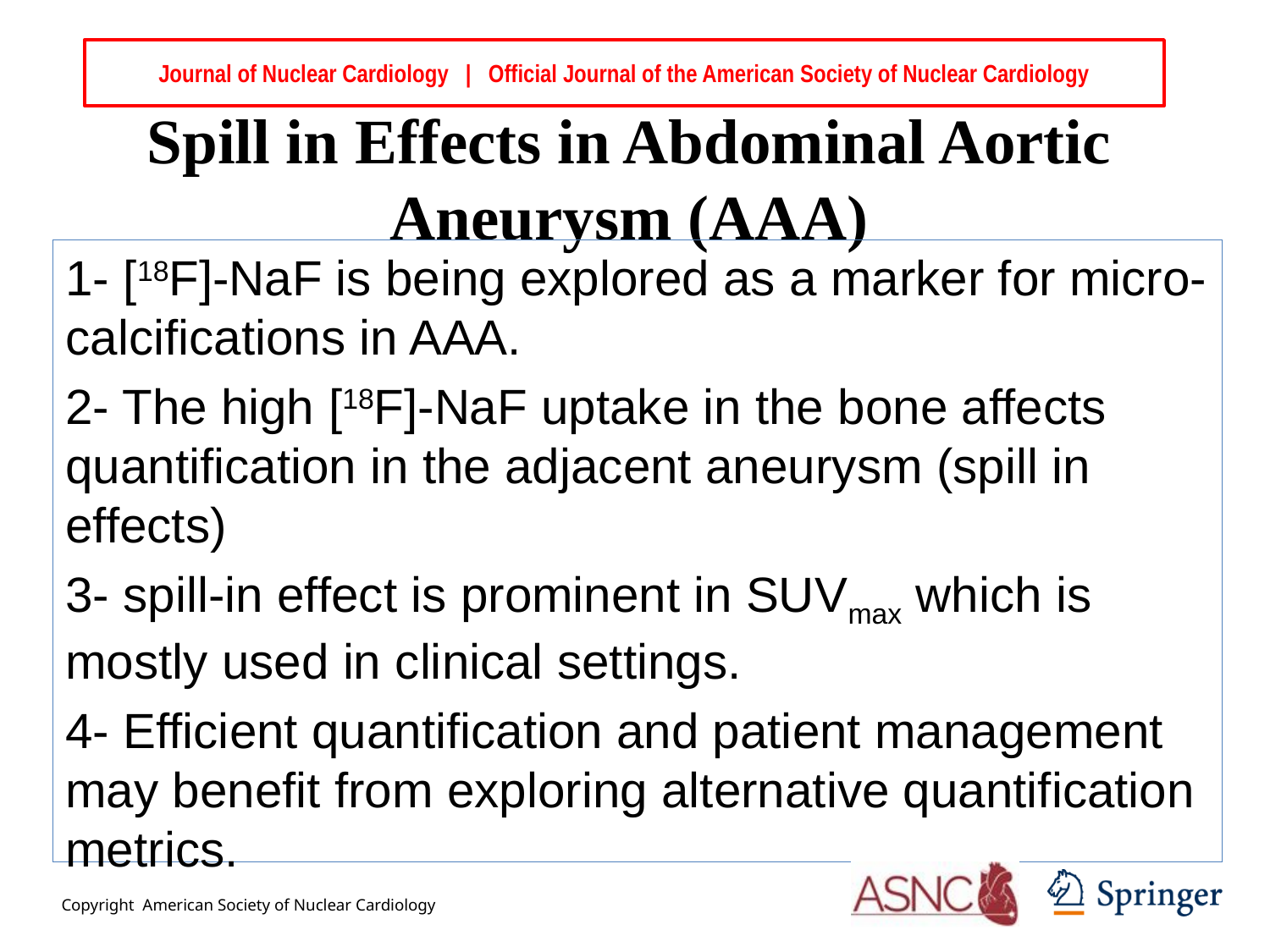

Journal of Nuclear Cardiology | Official Journal of the American Society of Nuclear Cardiology
# Spill in Effects in Abdominal Aortic Aneurysm (AAA)
1- [18F]-NaF is being explored as a marker for micro-calcifications in AAA.
2- The high [18F]-NaF uptake in the bone affects quantification in the adjacent aneurysm (spill in effects)
3- spill-in effect is prominent in SUVmax which is mostly used in clinical settings.
4- Efficient quantification and patient management may benefit from exploring alternative quantification metrics.
Copyright American Society of Nuclear Cardiology

## Slide 3
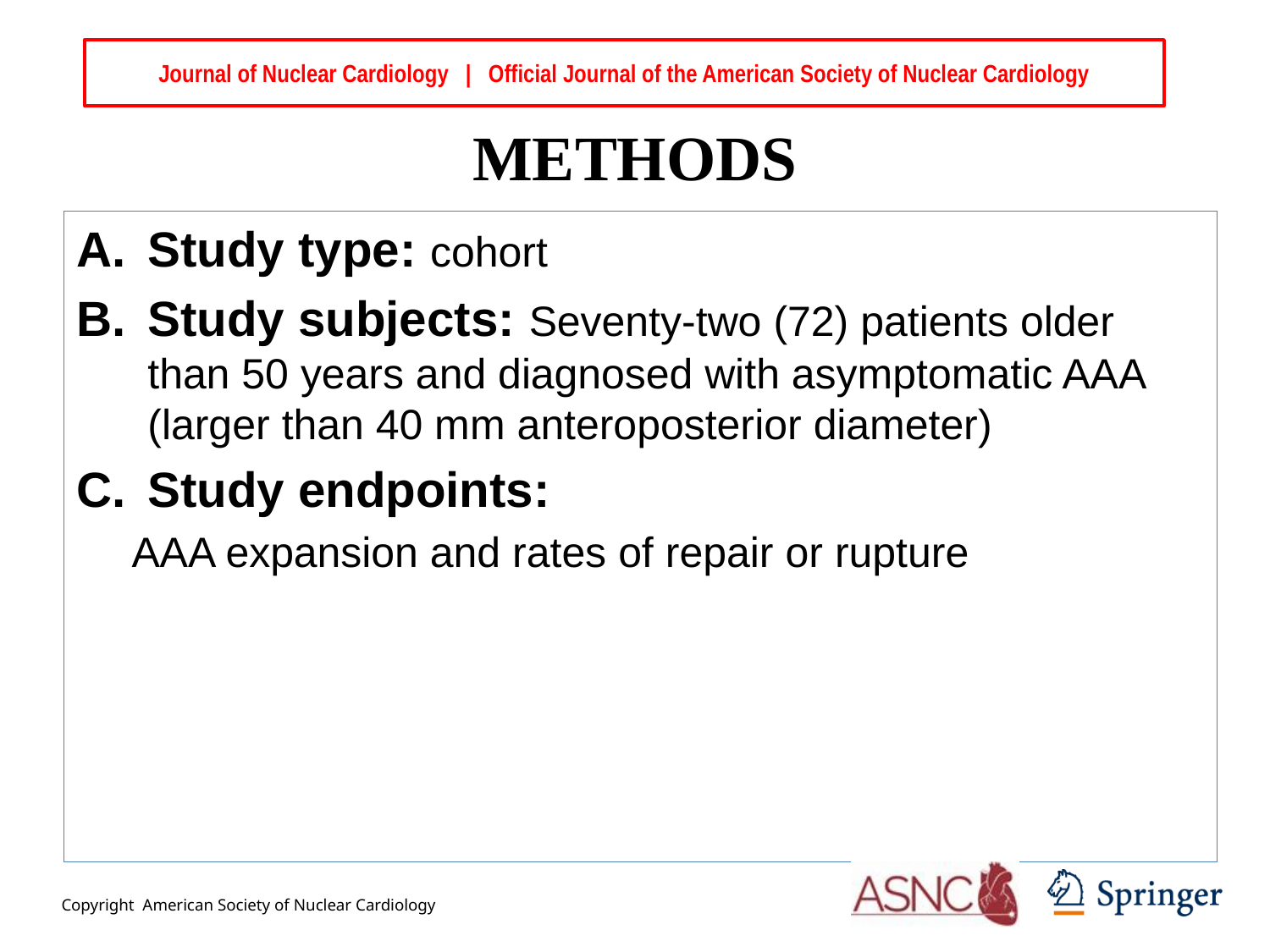

Journal of Nuclear Cardiology | Official Journal of the American Society of Nuclear Cardiology
# METHODS
Study type: cohort
Study subjects: Seventy-two (72) patients older than 50 years and diagnosed with asymptomatic AAA (larger than 40 mm anteroposterior diameter)
Study endpoints:
AAA expansion and rates of repair or rupture
Copyright American Society of Nuclear Cardiology

## Slide 4
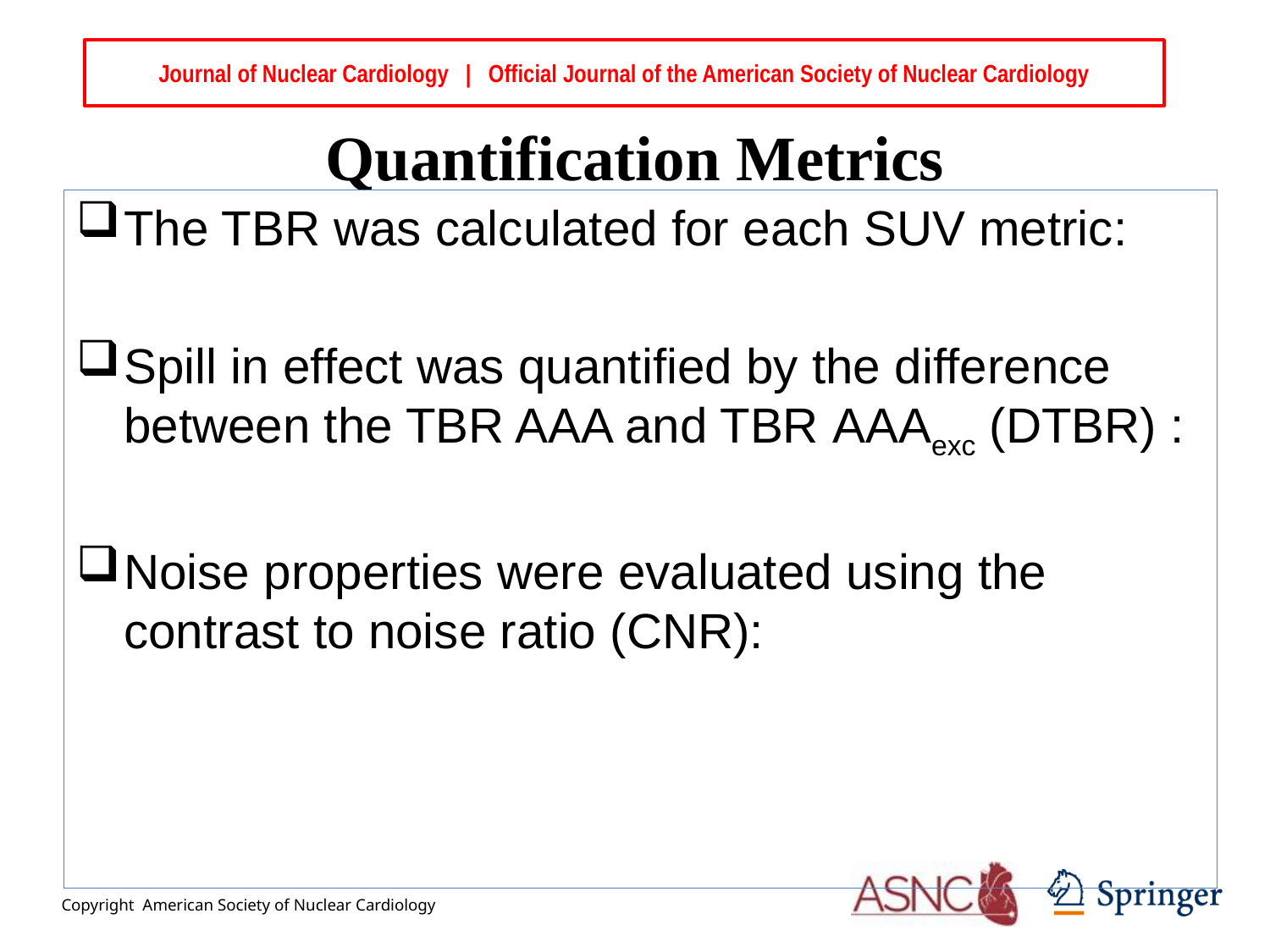

Journal of Nuclear Cardiology | Official Journal of the American Society of Nuclear Cardiology
# Quantification Metrics
Copyright American Society of Nuclear Cardiology

## Slide 5
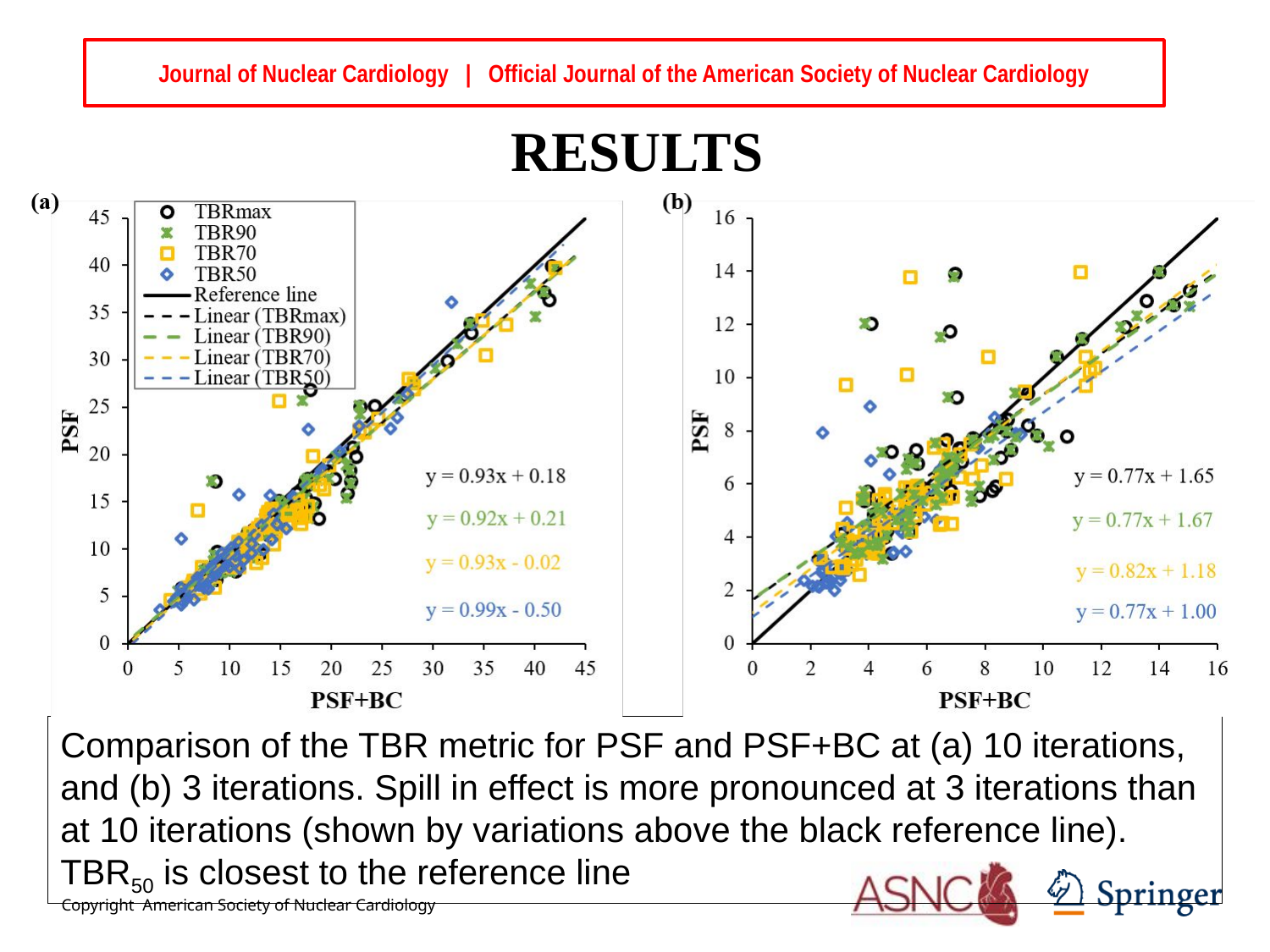

Journal of Nuclear Cardiology | Official Journal of the American Society of Nuclear Cardiology
# RESULTS
Comparison of the TBR metric for PSF and PSF+BC at (a) 10 iterations, and (b) 3 iterations. Spill in effect is more pronounced at 3 iterations than at 10 iterations (shown by variations above the black reference line). TBR50 is closest to the reference line
Copyright American Society of Nuclear Cardiology

## Slide 6
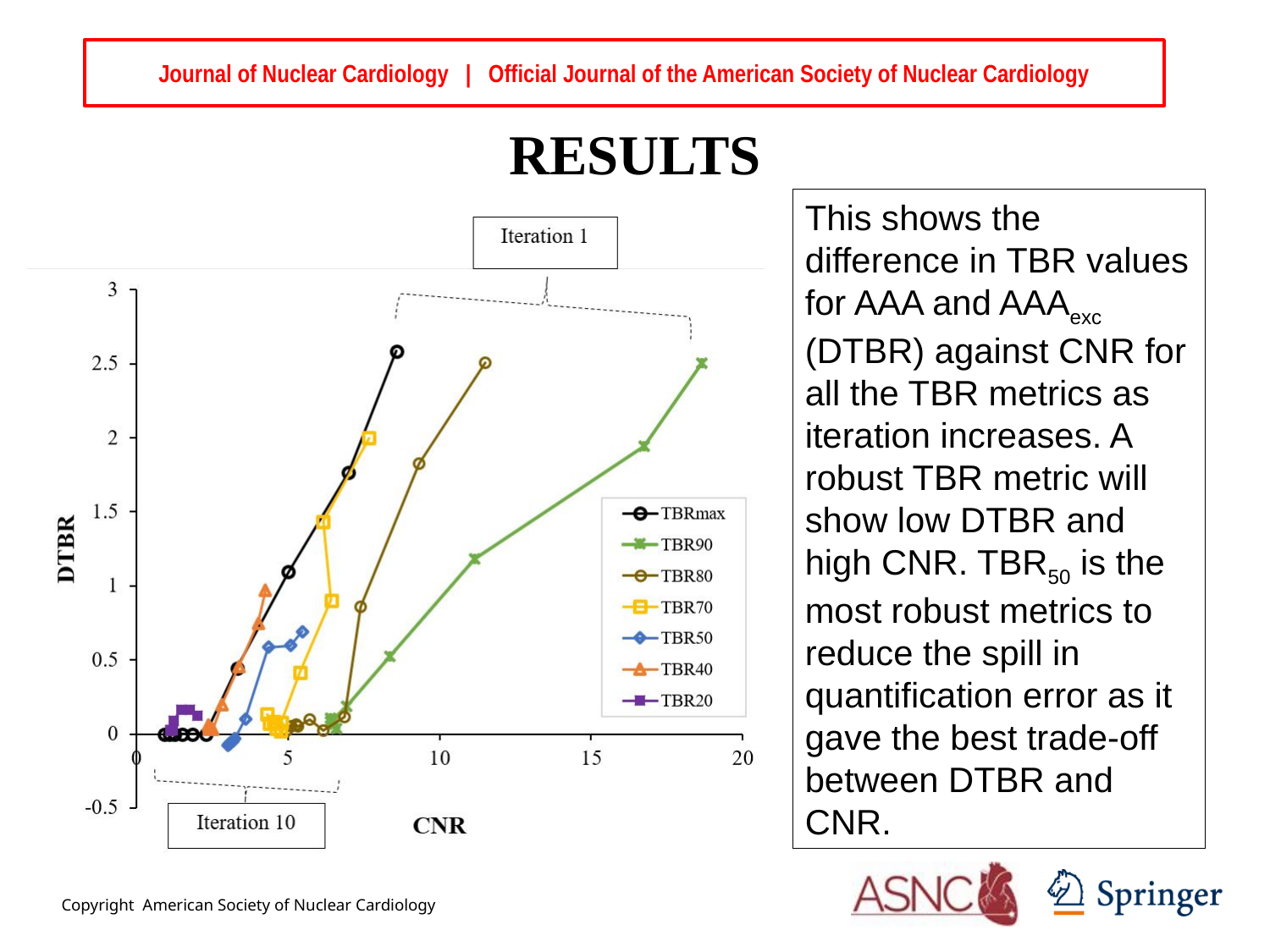

Journal of Nuclear Cardiology | Official Journal of the American Society of Nuclear Cardiology
# RESULTS
This shows the difference in TBR values for AAA and AAAexc (DTBR) against CNR for all the TBR metrics as iteration increases. A robust TBR metric will show low DTBR and high CNR. TBR50 is the most robust metrics to reduce the spill in quantification error as it gave the best trade-off between DTBR and CNR.
Copyright American Society of Nuclear Cardiology

## Slide 7
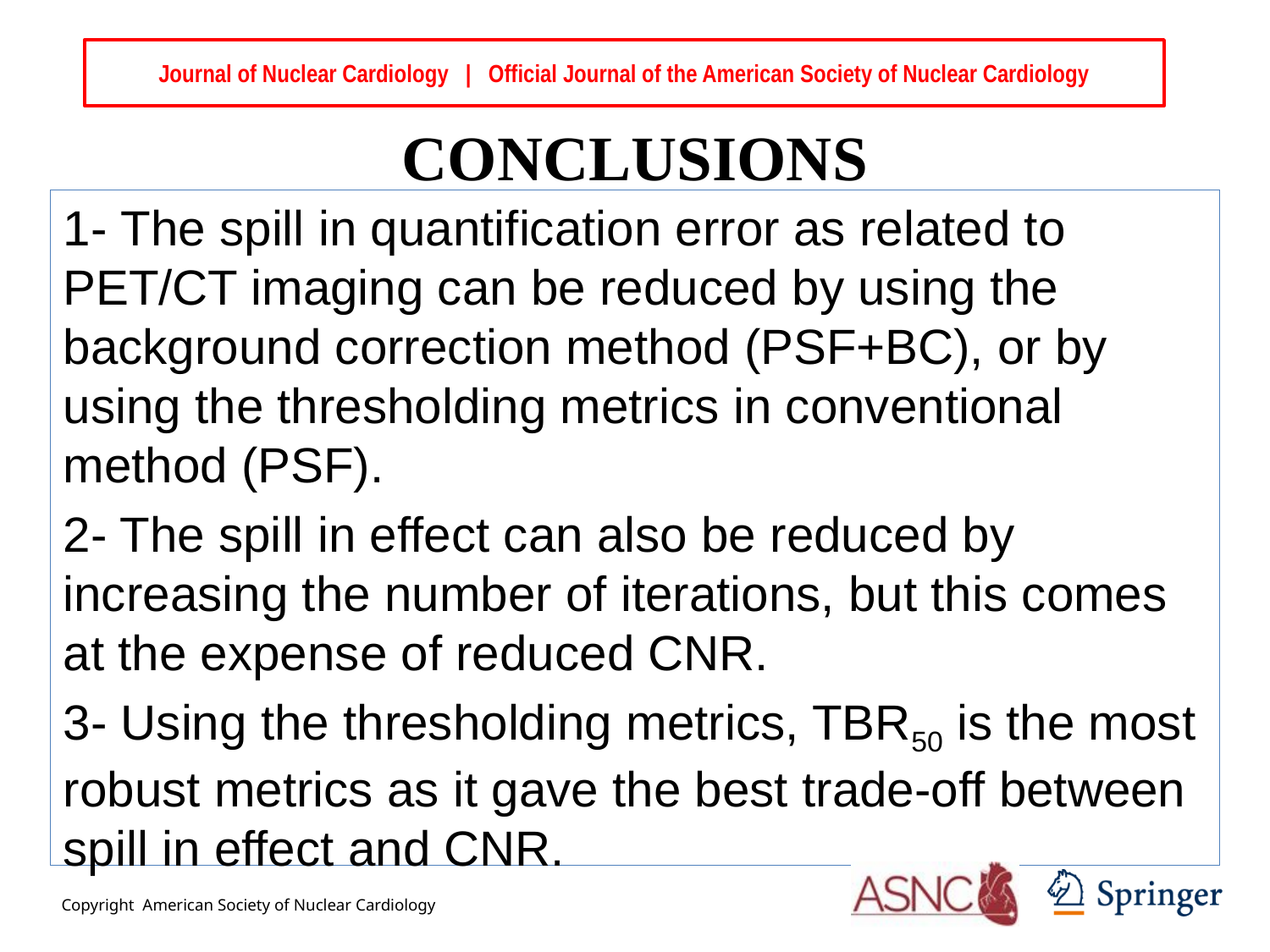

Journal of Nuclear Cardiology | Official Journal of the American Society of Nuclear Cardiology
# CONCLUSIONS
1- The spill in quantification error as related to PET/CT imaging can be reduced by using the background correction method (PSF+BC), or by using the thresholding metrics in conventional method (PSF).
2- The spill in effect can also be reduced by increasing the number of iterations, but this comes at the expense of reduced CNR.
3- Using the thresholding metrics, TBR50 is the most robust metrics as it gave the best trade-off between spill in effect and CNR.
Copyright American Society of Nuclear Cardiology
